# Circ-GLI1 promotes metastasis in melanoma through interacting with p70S6K2 to activate Hedgehog/GLI1 and Wnt/β-catenin pathways and upregulate Cyr61

**DOI:** 10.1038/s41419-020-02799-x

**Published:** 2020-07-30

**Authors:** Jun Chen, Xiaobo Zhou, Jie Yang, Qilin Sun, Yang Liu, Ningli Li, Zhen Zhang, Hui Xu

**Affiliations:** 1 Department of Dermatology and Dermatologic Surgery, Shanghai Ninth People’s Hospital, Affiliated to Shanghai Jiaotong University School of Medicine, Center for Specialty Strategy Research of Shanghai Jiaotong University China Hospital Development Institute, Shanghai, 200011 China; 2 Department of Plastic and Reconstructive Surgery, Shanghai Ninth People’s Hospital, Affiliated to Shanghai Jiaotong University School of Medicine, Center for Specialty Strategy Research of Shanghai Jiaotong University China Hospital Development Institute, Shanghai, 200011 China; 3 Core Facility of Basic Medical Sciences, Shanghai Jiaotong University School of Medicine, Shanghai, 200025 China; 4 Department of Immunology, Shanghai Jiaotong University School of Medicine, Shanghai, 200025 China

**Keywords:** Skin cancer, Cell biology

## Abstract

Circular RNAs (circRNAs) are emerging regulators in the development of human cancers. However, the role of circRNAs in melanoma is poorly understood. Microarray analysis and qRT-PCR was applied to screen out circRNAs that were differentially expressed in melanoma cells compared to normal cells. Currently, we first proved that inhibition of CYR61, an angiogenesis factor with controversial functions in melanoma, restrained cell migration, invasion and angiogenesis in melanoma. Thereafter, a novel circRNA hsa_circ_0027247 derived from GLI1 (circ-GLI1) was identified to positively modulate CYR61 expression in melanoma cell lines. Besides, silencing circ-GLI1 hindered melanoma cell metastasis as well. Interestingly, we unveiled that circ-GLI1 enhanced CYR61 transcription by an indirect manner. Meanwhile, circ-GLI1 activated Hedgehog/GLI1 and Wnt/β-catenin pathways by affecting the degradation of GLI1 and β-catenin. Moreover, we found that circ-GLI1 interacted with p70S6K2 to induce GSK3β phosphorylation at Ser9, and therefore blocked the binding of GSK3β with GLI1 and β-catenin so as to elevate their protein expression. Of note, CYR61 was transcriptionally activated by MYC, a well-recognized downstream target of both GLI1 and β-catenin. In conclusion, circ-GLI1 exacerbates the metastasis and angiogenesis of melanoma by upregulating Cyr61 via p70S6K2-dependent activation of Hedgehog/GLI1 and Wnt/β-catenin pathways.

## Background

Melanoma is the most malignant tumor that occurs in skin^1^ and has an increasing incidence in recent years^2^. Nowadays, melanoma remains the leading cause of skin cancers-related death owing to its high metastasis^3,4^. Despite the development of treatments^5,6^, the prognosis of patients with melanoma still disappointing^7,8^. Thus, discovering novel molecules and developing new effective therapeutic targets are of great significance to improve the prognosis in melanoma patients.

Cysteine-rich angiogenic inducer 61 (Cyr61) is a matricellular protein that belongs to CCN family (Cyr61, CTGF, and NOV)^9^. Recently, Cyr61 has been considered as an angiogenic factor which functions in different biological behaviors. Cyr61 has been validated to exert facilitating role in tumorigenesis of many cancer types, such as colorectal cancer^10^ and ovarian cancer^11^. However, several reports revealed that Cyr61 can act as a tumor suppressor in lung cancer^12^ and hepatocellular carcinoma^13^. Intriguingly, the role of Cyr61 in melanoma is still unclear. Some reports indicated that Cyr61 could play a tumor-suppressive role in melanoma^14,15^. On the other hand, Cyr61 was highly expressed in highly aggressive or metastatic melanoma^16–18^. Based on these evidences, we supposed that the function of Cyr61 in melanoma might alter along with the tumor progression. Therefore, this study aimed at investigating the role of Cyr61 in melanoma metastasis.

Non-coding RNAs (ncRNAs) have attracted more and more attention due to their sophisticated regulatory roles in human diseases^19,20^. Circular RNAs (circRNAs) are a new class of ncRNA transcripts that are regarded as novel regulators in the pathogenesis of multiple diseases, including cancers^21^. Besides, some reports have strongly demonstrated the involvement of circRNAs in melanoma development. For example, a novel circRNA called hsa_circ_0025039 plays a promotion role in melanoma cell growth, invasion and glucose metabolism^22^. Luan et al.^23^ identified circRNA_0084043 as a tumor promotor in malignant melanoma^23^. However, the role of circRNAs in melanoma remains to be unmasked.

In the present study, we uncovered that hsa_circ_0027247, a novel circRNA annotated to GLI1 gene (called as circ-GLI1), was highly expressed in melanoma cells. This study focused on the role of circ-GLI1 in melanoma metastasis and its correlation with Cyr61.

## Materials and methods

### Cell lines and reagents

Human epidermal melanocyte (HEMa-LP) and six melanoma cells (A375, MEL-RM, B16, M14, SK-MEL-2, and SK-MEL-28), human embryonic kidney cell (HEK293T), and human umbilical vein endothelial cells (HUVECs) were all obtained from American Type Culture Collection (ATCC; Manassas, VA, USA). DMEM (Invitrogen, Carlsbad, CA, USA) with 10% fetal bovine serum (FBS; Invitrogen) and 1% antibiotics (Invitrogen) was used for cell culture in 5% CO_2_/95% air at 37 °C. All reagents used in our study, including GDC-0449 (Hedgehog inhibitor), XAV-939 (Wnt/β-catenin inhibitor), cycloheximide (CHX; protein synthesis inhibitor), MG132 (proteasome inhibitor), SAG (Hedgehog activator), and LiCl (Wnt/β-catenin activator), Cyclopamine (Hedgehog inhibitor), and DKK1 (Wnt/β-catenin inhibitor), were purchased from Sigma-Aldrich (St. Louis, MO, USA).

### qRT‐PCR

Total RNAs were extracted from A375 or B16 cells using TRIzol Reagent (Invitrogen) and then reversely transcribed to first-stand cDNA. qRT‐PCR was run with SYBR^®^ Premix Ex Taq™ II kit (Takara, Tokyo, Japan) on 7500 Real‐Time PCR System (Applied Biosystems, Foster City, CA, USA). Results calculated by 2^−ΔΔCt^ method were normalized to GAPDH. All primers used in this experiment were listed in Supplementary Table 1.

### Transfection

The sh-Cyr61#1/2/3, sh-circ-GLI1#1/2 and sh/MYC transfection plasmids were separately constructed utilizing their specific short hairpin RNAs (shRNAs; GenePharma, Shanghai, China) sequences, with non-specific shRNAs as control (shCtrl). The pcDNA3.1 (+) CircRNA Mini Vector targeting circ-GLI1, pcDNA3.1 expressing p70S6K2, MYC or Cyr61 was simultaneously generated at GenePharma, with empty vector as control. Transfection in A375 or B16 cells was performed with Lipofectamine 2000 (Invitrogen). Transfected cells were subjected to qRT-PCR analysis after 48 h. All samples were assayed in triplicate.

### Sanger sequencing

The hsa_circ_0027247 (circ-GLI1) sequence was obtained using divergent primers sent to Sangon (Shanghai, China) for Sanger sequencing analysis.

### Wound-healing assay

Scratch wound-healing assay was conducted to measure the migratory ability of indicated melanoma cells. In brief, A375 and B16 cells transfected with indicated plasmids for 48 h. Cells were cultured in 6-well plates at a density of 5 × 10^5^ cells per well and incubated overnight. After washing twice with PBS, cells were added in the serum free medium. Wounds were observed at 0 h and 24 h after the scratching. Images were obtained under an inverted microscope (Nikon, Tokyo, Japan) at a magnification of ×200. Results were analyzed with Image-Pro Plus software (Media Cybernetics, Inc., Rockville, MD, USA). All samples were assayed in triplicate.

### Transwell assay

Transfected A375 or B16 cells were planted in 24-well plates at a density of 1 × 10^5^ cells per well. Cells were put into the upper chamber coated with or without Matrigel-coated membrane (BD Bioscience, Bedford, MA, USA). The lower chambers were added into medium containing 10% FBS as chemoattractant. Cells migrated or invaded across the membrane were stained with 0.2% crystal violet solution for 30 min and captured under a light microscope (Olympus). All samples were assayed in triplicate.

### Tube formation assay

After 48 h of transfection, the A375 and B16 cells were centrifuged, and the supernatant was collected. Tumor-conditioned medium was obtained by adding the mixture of A375 or B16 cell supernatant, DMEM medium and FBS at a ratio of 4:5:1. Each well of a 96-well plate was added with Matrigel (50 μL) and incubated for 30 min in a 37 °C incubator. After coagulation, tumor-conditioned medium and HUVEC suspension were co-cultured in the Matrigel-coated plate for 8 h at 37 °C with 5% CO_2_. The number of formed small tubes was counted, and images were obtained from four randomly selected fields under a phase contrast microscope. All samples were assayed in triplicate.

### Angiogenesis assay on chicken chorioallantoic membrane (CAM)

Day-8 fertilized chicken eggs were acquired from Yueqin Breeding (Guangdong, China) for CAM assay. The air sac was generated. The small window was cut in the shell. A375 or B16 cell culture medium resuspended in sterile filter paper was put onto the CAM. Five days of implantation, paper was photographed and then the number of branches was calculated. All samples were assayed in triplicate.

### Microarray analysis

The global profile of human circRNAs was analyzed in HEMa-LP and six melanoma cells by Arraystar Human circRNA Microarray v.2.0 (Arraystar, Rockville, MD, USA).

### Nucleic acid electrophoresis

Through TE buffer AGAR gels from Thermo Fisher Scientific (Waltham, MA, USA), cDNA and gDNA PCR products of circ-GLI1 were tested. As the DNA markers, DL600 (KeyGen, Nanjing) was adopted. Electrophoretic voltage (110V) was performed to isolate DNA. Ultraviolet radiation was applied to determine results. All samples were assayed in triplicate.

### Dual-luciferase reporter assay

Cyr61 promoter was sub-cloned into pGL3-Basic luciferase reporter vector (Promega, Madison, WI, USA) and then transfected into cells with indicated transfection plasmids or reagents. The promoter sequences of Cyr61 was obtained from Ensembl (http://asia.ensembl.org/index.html) and provided in Supplementary Table 1. The pGL3 vector containing Cyr61 promoter sequence was co-transfected with shCtrl, sh/circ-GLI1 or sh/circ-GLI1 + pcDNA3.1/p70S6K2 into HEK293T cells that were seeded into 24-well plates at a density of 2 × 10^4^ cells per well. Forty-eight hours later, Dual-Luciferase Report Assay System (Promega) was used to monitor luciferase activity. For TOP/FOP-Flash analysis, HEK-293T cells were co-transfected with TOP/FOP Flash (Genechem, Shanghai, China) together with shCtrl, sh/circ-GLI1 or sh/circ-GLI1 + pcDNA3.1/p70S6K2. All samples were assayed in triplicate.

### Evaluation of GLI1 regulatory reporter gene by β-lactamase assay

GLI1 regulatory reporter gene activity was evaluated by β-lactamase assay. Transfection of the pLenti-bsd/GLI-bla vectors (Invitrogen) A375 and B16 cells was finished by Lipofectamine reagent (Invitrogen). The vectors contained the β-lactamase reporter gene under a transcriptional control of the (8×) Gli response element with the tata-minimal promoter. The transfected cells were subsequently cultured in the growth medium containing 3.8 μg/ml blasticidin for about 2 weeks to establish stable cell lines. Several stable clones were transfected with sh-GLI1 and β-lactamase activity in a few clones were validated to be reduced by more than 70% upon GLI1-silencing. β-lactamase activity was quantitated with application of GeneBLAzer™ Detection Kits (Invitrogen) in reference to the manufacturer’s instructions. After the 6× substrate loading solution was added to the cells with 1 × final concentration, cells in the buffer underwent incubation for 6 h. β-lactamase activity was thereafter measured by applying a fluorescent plate reader. All samples were assayed in triplicate.

### RNA pull-down and mass spectrometry assay

Biotin-labeled RNAs were separately in vitro transcribed, purified and incubated with cell lysates. At length, the pulled-down complex was analyzed by qRT-PCR or run on SDS-PAGE and mass spectrometry.

### Western blotting

The extracted total protein was subjected to SDS-PAGE and transferred onto PVDF membranes (Millipore, Bedford, MA, USA). Following blockade, membranes were treated with primary antibodies overnight and with secondary antibody for 2 h in the dark. Primary antibodies against GLI1 (ab49314), SOX2 (ab93689), MYC (ab9106), VEGF (ab32152), β-catenin (ab32572), MMP2 (ab215986), p70S6K2 (ab184551), GSK3β (ab93926), p-GSK3β (Y216) (ab75745), p-GSK3β (S9) (ab75814), Cyr61 (ab24448), and GAPDH (ab8245), were all from Abcam (Cambridge, MA, USA). All samples were assayed in triplicate.

### Fluorescent in situ hybridization (FISH)

Using fluorescent in situ hybridization kit (RiboBio), RNA FISH were carried out. The circ-GLI1 probes were labeled with Cy3 fluorescent dye (Sigma-Aldrich). Fluorescence signals were detected by confocal laser-scanning microscope (Olympus, Tokyo, Japan). All samples were assayed in triplicate.

### RNA immunoprecipitation (RIP)

Using Imprint RNA immunoprecipitation kit (Millipore), RIP assay was performed. Cells in RIP lysis buffer were immunoprecipitated with antibodies against p70S6K2 (1:200), with IgG (1: 200) as negative control, followed by magnetic beads. Purified RNA was evaluated through qRT-PCR or western blot. All samples were assayed in triplicate.

### Co-immunoprecipitation (Co-IP)

Cells were lysed in IP Lysis (Thermo Fisher Scientific). Using GSK3β or IgG antibody, the immune complex was prepared and subjected to Pierce™ Classic IP Kit (Thermo Fisher Scientific). Samples were separated on SDS-PAGE and detected by western blotting. All samples were assayed in triplicate.

### Chromatin immunoprecipitation (ChIP)

After treatment with 4% formaldehyde, DNA-protein cross-links were sonicated into 200–500 bp chromatin fragments and immunoprecipitated with MYC or IgG-specific antibody. The final precipitated chromatin was assayed by qRT-PCR. All samples were assayed in triplicate.

### In vivo experiments

To monitor metastasis in vivo, 2 × 10^5^ A375 cells transfected with shCtrl, sh/circ-GLI1#1, or sh/circ-GLI1#1 + Cyr61, were injected respectively from the tail vain into the 4-week-old male BALB/c nude mice (Institute of Zoology, China Academy of Sciences). Mice were sacrificed following 32 days of injection and tissue sections underwent hematoxylin and eosin (HE) staining. The metastatic nodules were monitored by an inverted microscope (Nikon, Japan).

To monitor the angiognenesis in vivo, A375 cells with indicated injection were subcutaneously injected into nude mice and after 4 weeks, tissue sections from sacrificed mice were subjected to immunohistochemistry analysis of CD31 to measure the vascular density. In brief, the collected xenografts were embedded in paraffin and sliced into sections of 4-μl thickness. Thereafter, sections underwent incubation with anti-CD31 at 4 °C for 12 h, and then the incubation followed by secondary antibodies. Later, the tissue sections were monitored applying the inverted microscope (Nikon, Japan) at ×200 magnification. In vivo study was approved by the Ethic committee of Shanghai Ninth People’s Hospital, Affiliated to Shanghai Jiaotong University School of Medicine.

### Statistical analysis

GraphPad Prism 6.0 (GraphPad Software, La Jolla, CA, USA) was used for statistical analysis. All experiments were run in triplicate. Data were shown as mean ± standard deviation (SD). Student’s *t*-test or one-way ANOVA was utilized to examine statistical difference, with the significant level of *p* < 0.05.

## Results

### Cyr61 contributes to cell migration, invasion, and angiogenesis in melanoma

**Fig. 1:**
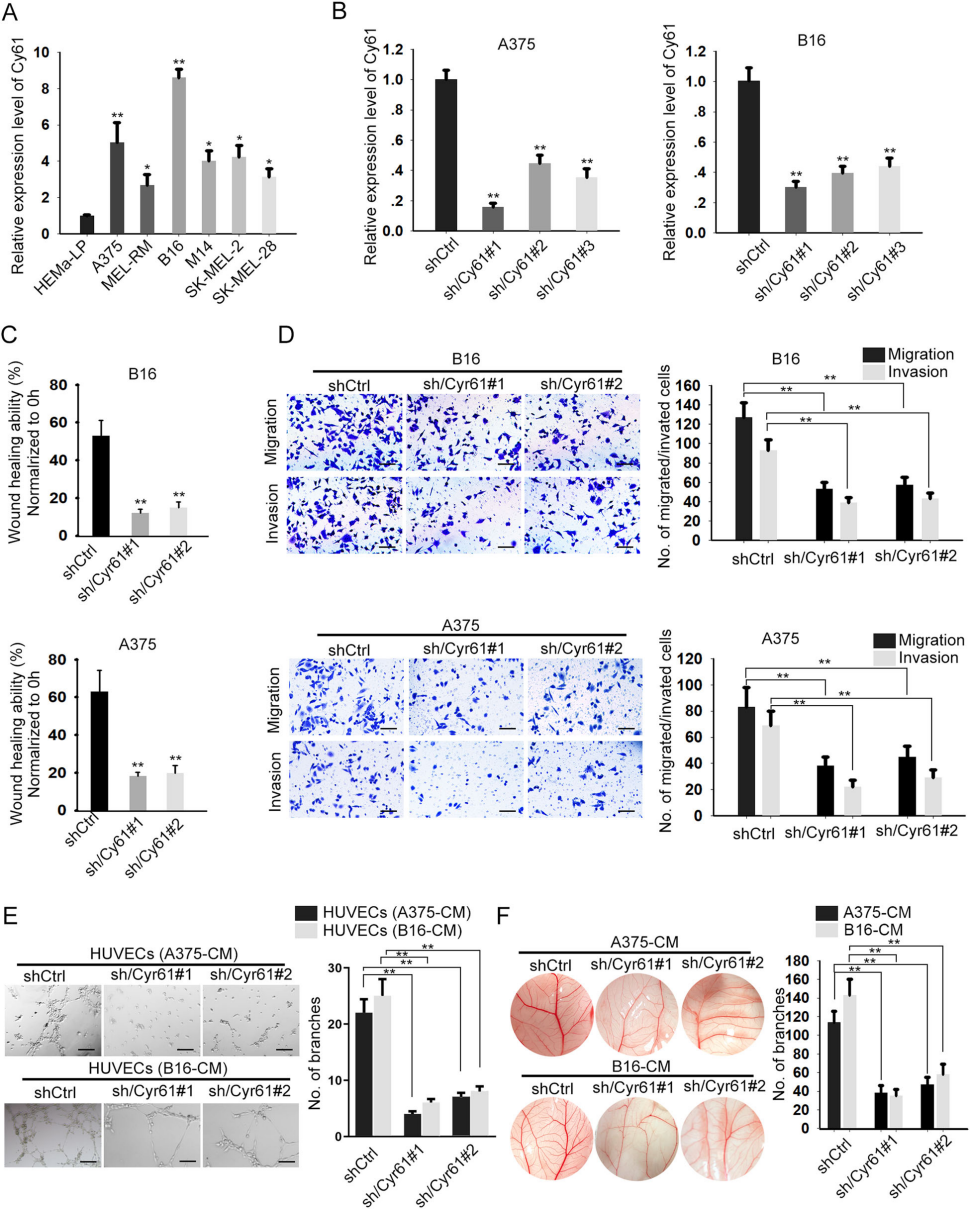
Cyr61 depletion suppresses melanoma metastasis. **a** Cyr61 was highly expressed in melanoma cells, compared to human epidermal melanocytes HEMa-LP. One-way ANOVA. **b** Cyr61 expression was silenced in A375 and B16 cells using specific shRNAs. Results were detected after 48 h’ transfection. One-way ANOVA. **c**, **d** Forty-eight hours after transfection, wound-healing and transwell assays indicated that Cyr61 depletion obviously weakened the migratory and invasive abilities of A375 and B16 cells. Scale bar = 200 μm. One-way ANOVA. **e** Forty-eight hours after transfection, HUVECs were cultured in CM of melanoma cells with indicated transfection. Tube formation assays were used to detect the tube formation in indicated cells. Scale bar = 200 μm. One-way ANOVA. **f** Chicken chorioallantoic membrane (CAM) assays revealed the blood vessels in response to Cyr61 knockdown. One-way ANOVA. All data were obtained from at least three replicates and shown as mean ± SD. ^*^P < 0.05 and ^**^P < 0.01.

First, we tested the expression pattern of Cyr61 in melanoma cell lines. As indicated in Fig. 1a, the expression level of Cyr61 was elevated in melanoma cells compared to the normal HEMaLP cells. On this basis, we silenced Cyr61 expression in A375 and B16 cells which expressed the highest level of Cyr61. As validated by qRT-PCR, the expression of Cyr61 was apparently inhibited in both A375 and B16 cells under the transfection of three Cyr61-specific shRNAs for 48 h (Fig. 1b), and cells transfected with shCyr61#1 and shCyr61#2 were used for subsequent experiments due to their relative higher knockdown efficiency. Moreover, loss of Cyr61 expression markedly hindered wound-healing ability of both A375 and B16 cells (Fig. 1c). Also, transwell assays indicated that the migratory and invasive abilities of the two melanoma cells were both abrogated upon Cyr61 inhibition (Fig. 1d). In addition, tube formation and CAM assays suggested that silencing of Cyr61 in B16 and A375 cells obviously hampered the angiogenesis (Fig. 1e, f). Collectively, these data uncovered that Cyr61 contributes to cell motility and angiogenesis in melanoma.

### Circ-GLI1 plays a facilitating role in melanoma cell migration and angiogenesis through targeting Cyr61

**Fig. 2:**
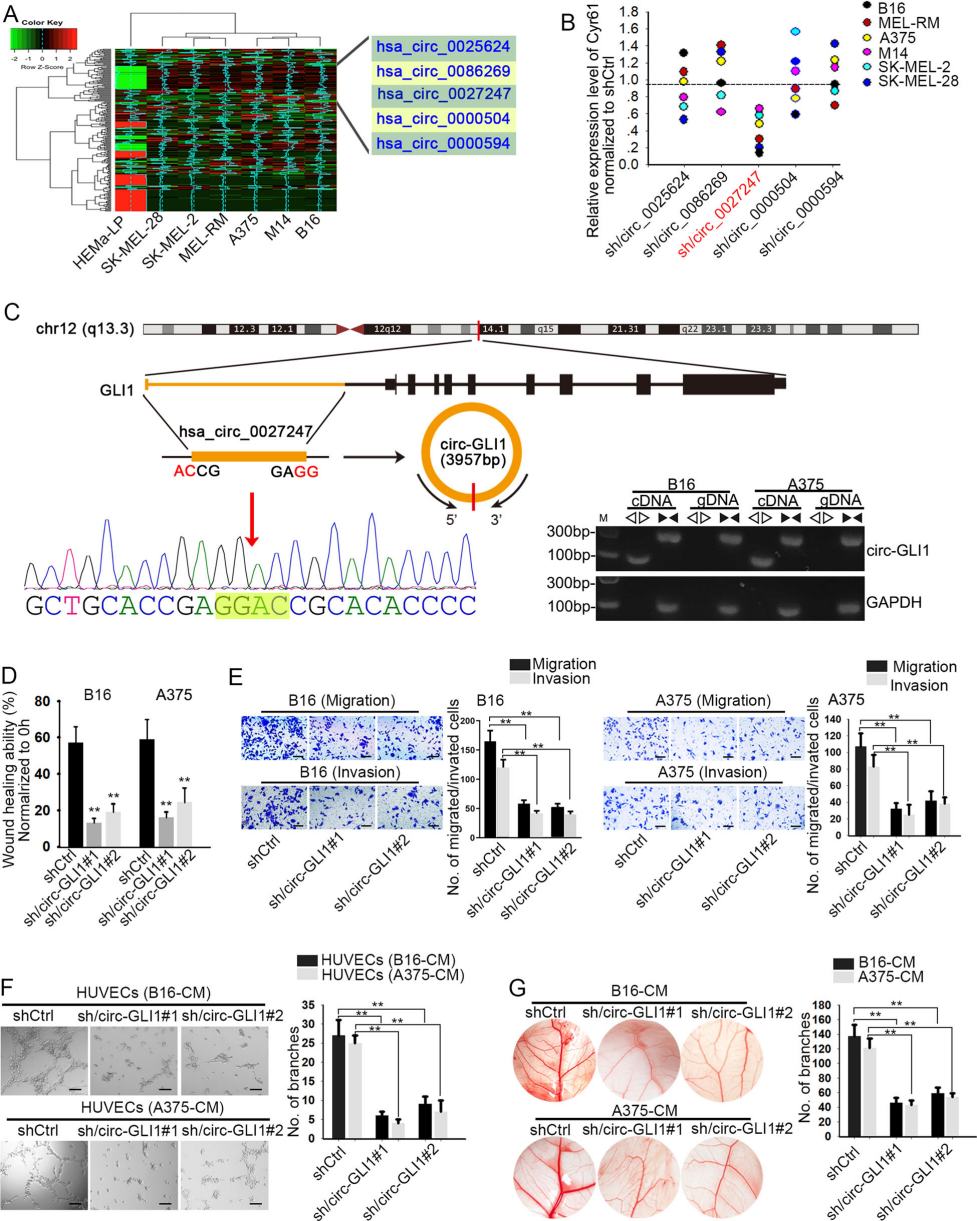
Circ-GLI1 promotes melanoma metastasis. **a** Five most upregulated circRNAs were screened out by microarray analysis. **b** Cyr61 expression was reduced responding to sh/circ_0027247 in melanoma cells. Results were detected at 48 h after transfection. One-way ANOVA. **c** The genomic location of circ_0027247 screened from UCSC was illustrated. The circular structure of circ_0027247 (circ-GLI1) was identified by Sanger sequencing and agarose gel electrophoresis. **d**, **e** Forty-weight hours after transfections, wound-healing and transwell assays proved that the depleted circ-GLI1 lessened cell migration and invasion. Scale bar = 200 μm. One-way ANOVA. **f** Forty-weight hours after transfections, tube formation assay validated circ-GLI1 depletion repressed angiogenesis in vitro. Scale bar = 200 μm. One-way ANOVA. **g** CAM assays revealed the angiogenesis in the chicken chorioallantoic membrane after silencing of circ-GLI1. One-way ANOVA. All data were obtained from at least three replicates and shown as mean ± SD. ^**^P < 0.01.

Recently, circRNAs have been emerged as new regulators in cancer development. Herein, we wondered whether there were certain circRNAs in the upstream of Cyr61 in melanoma. After analyzing genes with differential expression in melanoma cells compared to the normal HEMaLP cells, we screened out five circRNAs that were remarkably upregulated in six melanoma cells (Fig. 2a). Importantly, we observed that only the inhibition of hsa_circ_0027247 resulted in a distinct reduction on Cyr61 expression in six melanoma cells (Fig. 2b). Furtherly, the circular structure of hsa_circ_0027247 (a circRNA that derived from GLI1 gene, which was subsequently named circ-GLI1) was illustrated according to the genomic location in UCSC (Fig. 2c, upper). The circular structure and the back-spliced region of circ-GLI1 was further confirmed by Sanger sequencing (Fig. 2c, left bottom). The specific amplification of circular transcripts in only cDNA by the use of divergent primers and that of linear transcript in both cDNA and gDNA by convergent primers (Fig. 2c, right bottom). Hence, we suspected that circ-GLI1 (hsa_circ_0027247) might play a role in melanoma through regulating Cyr61.

Thereafter, we attempted to investigate the precise function of circ-GLI1 in melanoma. Circ-GLI1 was silenced in melanoma cells by sh/circ-GLI1#1 and sh/circ-GLI1#2. The level of circ-GLI1 was decreased obviously at 48 h after transfection (Supplementary Fig. 1a). Later, we proved that inhibition of circ-GLI1 significantly impeded wound-healing ability in both A375 and B16 cells (Fig. 2d). Meanwhile, the migration and invasion were also dramatically declined in circ-GLI1-silenced A375 and B16 cells compared to the control group (Fig. 2e). More importantly, we observed that circ-GLI1 knockdown in melanoma cells pronouncedly retarded the formation of vascular branches, indicating that circ-GLI1 depletion hampered angiogenic process (Fig. 2f, g). Taken together, these findings unveiled that circ-GLI1 aggravates cell migration and angiogenesis in melanoma possibly through targeting Cyr61.

### Cyr61 mediates circ-GLI1-facilitated progression in melanoma

**Fig. 3:**
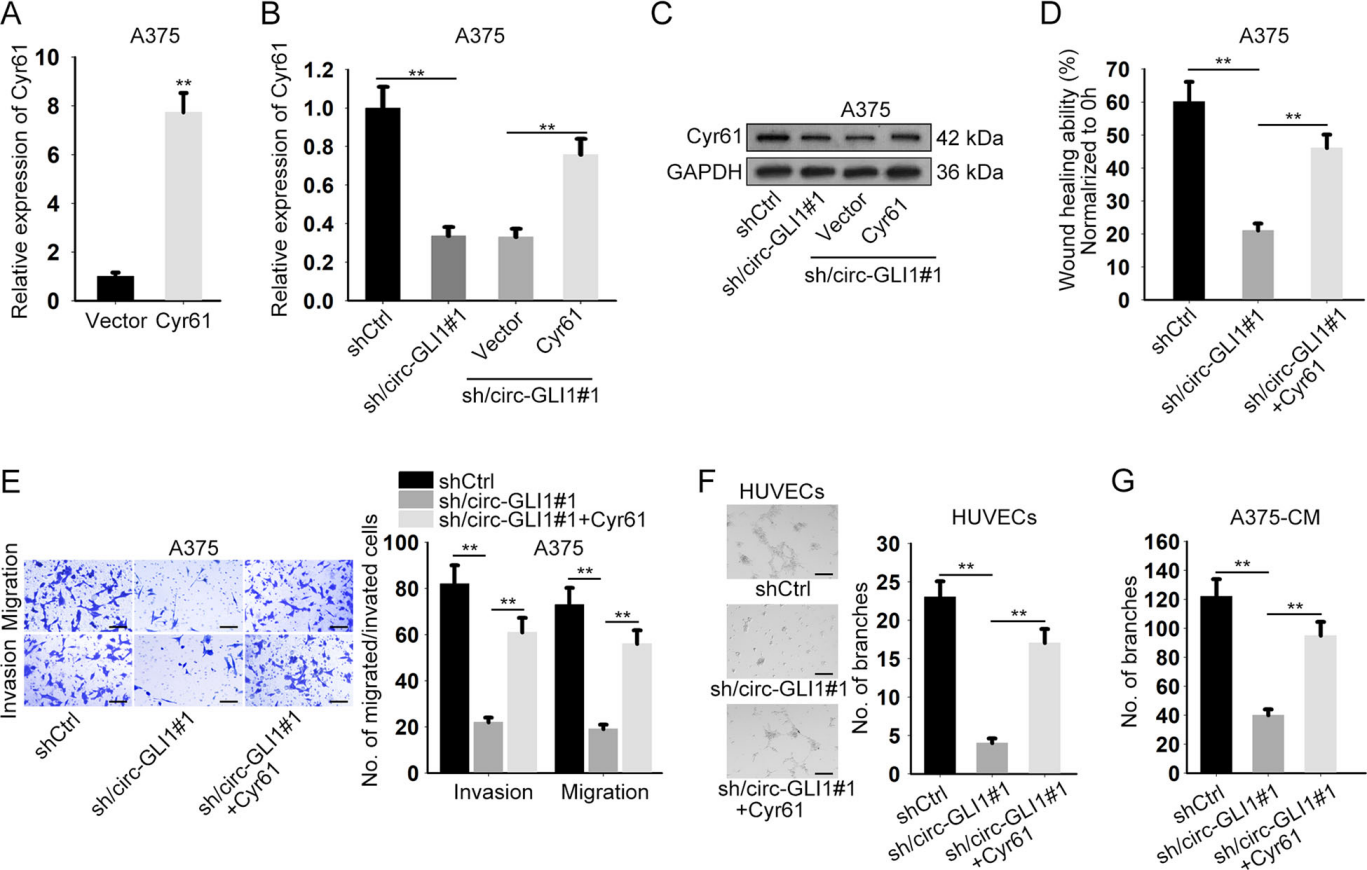
Circ-GLI1 promotes melanoma metastasis through Cyr61. **a** Cyr61 was overexpressed in A375 cells by transfecting with Cyr61 expression vector. Results were measured at 48 h after transfection by qRT-PCR analysis. Student’s t test. **b**, **c** The mRNA or protein level of Cyr61 in A375 cells under different conditions was respectively tested by qRT-PCR or western blot at 48 h after transfection. One-way ANOVA. **d**–**g** Forty-weight hours after transfection, wound-healing (Scale bar = 200 μm; one-way ANOVA), transwell (Scale bar = 200 μm; one-way ANOVA), tube formation (Scale bar = 200 μm; one-way ANOVA) and CAM assays (One-way ANOVA) were performed to evaluate the impact of CYR61 on cell migration, invasion and angiogenesis in A375 cells under circ-GLI1 depletion. All data were obtained from at least three replicates and shown as mean ± SD. ^**^P < 0.01.

To verify whether circ-GLI1 exerted functions in melanoma in a Cyr61-dependent manner, we conducted rescue assays in circ-GLI1-silenced A375 cells. Before rescue assays, Cyr61 was overexpressed by transfecting with Cyr61 expression vector. Forty-eight hours later, qRT-PCR analysis measured the transfection efficiency (Fig. 3a). As illustrated in Fig. 3b, c, co-transfection of pcDNA3.1/Cyr61 neutralized the inhibitory effect of circ-GLI1 knockdown on the mRNA and protein levels of Cyr61 in A375. Of importance, it was proven that Cyr61 overexpression recovered the wound-healing ability which was abated in circ-GLI1-silenced melanoma cells (Fig. 3d). Likewise, Cyr61 upregulation reversed the decreased migration and invasion of A375 cells caused by circ-GLI1 depletion (Fig. 3e). Moreover, we also explained that enhanced level of Cyr61 offset the restraining effect of circ-GLI1 suppression on angiogenesis (Fig. 3f, g). In sum, these findings proved that circ-GLI1 contributes to melanoma metastasis and angiogenesis via regulating Cyr61.

### Circ-GLI1 regulates Cyr61 through Hedgehog/GLI1 and Wnt/β-catenin pathway

**Fig. 4:**
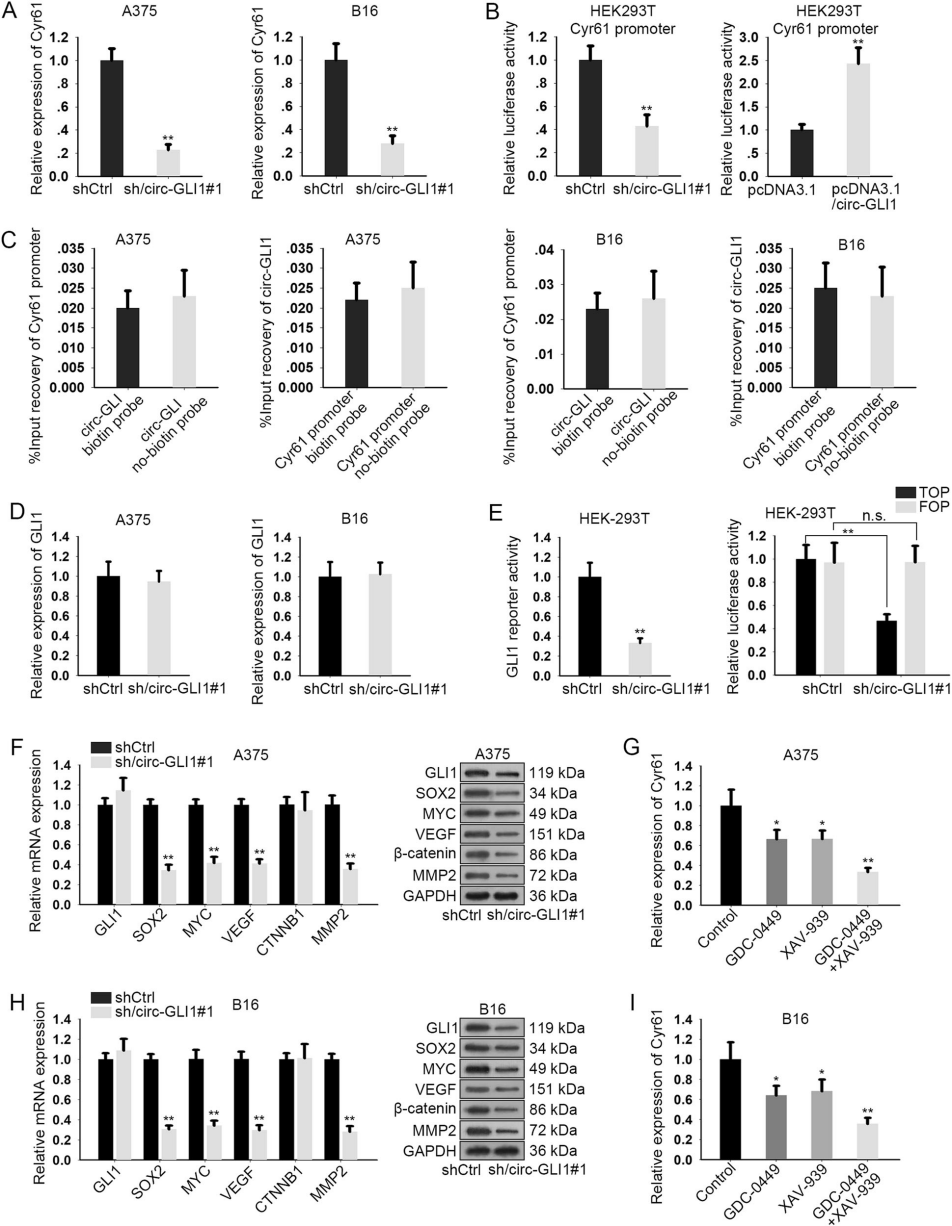
Circ-GLI1 regulates Cyr61 through Hedgehog and Wnt/beta-catenin pathways in melanoma. **a** qRT-PCR analysis revealed that circ-GLI1 knockdown led to the reduction of Cyr61 expression. Student’s t test. **b** Luciferase reporter assay was conducted to evaluate THE luciferase activity of Cyr61 promoter under circ-GLI1 upregulation or downregulation. Student’s *t* test. **c** The relative enrichment of Cyr61 promoter or circ-GLI1 in the pulled-down complex by circ-GLI1 biotin probe or Cyr61 promoter biotin probe. Student’s *t* test. **d** qRT-PCR result of GLI1 expression in melanoma cells with or without circ-GLI1 silence. Student’s *t* test. **e** GLI1 reporter activity or TOP luciferase activity was diminished by transfection with sh/circ-GLI1. Student’s *t* test. **f**–**h** The mRNA and protein levels of GLI1, SOX2, MYC, VEGF, CTNNB1, and MMP2 in A375 and B16 cells after silencing circ-GLI1. Student’s *t* test. **g**–**i** GDC-0449, XAV-939 or both weakened Cyr61 expression in A375 and B16 cells. All data were obtained from at least three replicates and shown as mean ± SD. One-way AVOVA. ^*^P < 0.05 and ^**^P < 0.01.

Furtherly, we wondered how circ-GLI1 modulated Cyr61 expression in melanoma. Since sh/circ-GLI1#1 had relative higher efficiency in inhibiting migration, invasion and angiogenesis, we chose it for subsequent assays. First, we verified that Cyr61 mRNA was distinctly downregulated in A375 and B16 cells in response to circ-GLI1 depletion (Fig. 4a). In addition, the activity of Cyr61 promoter was assessed in HEK293T cells after silencing or overexpression of circ-GLI1. The results of luciferase reporter assay indicated that the activity of Cyr61 promoter was decreased by circ-GLI1 silence but increased by circ-GLI1 downregulation (Fig. 4a), suggesting that circ-GLI1 regulated Cyr61 at transcriptional level. However, Cyr61 was not enriched in the pulldown of circ-GLI1 biotin probe and vice versa, suggesting that there was no direct interaction between circ-GLI1 and Cyr61 promoter (Fig. 4c). Hence, we supposed that circ-GLI1 indirectly modulated Cyr61 transcription in melanoma cells.

Increasing reports indicated that circRNAs could exert their function in cancer development through regulating the expression of the adjacent genes, especially their host genes^24^. GLI1 is widely recognized as the pivotal effector in Hedgehog signaling^25^. Hence, we suspected that circ-GLI1 might function in melanoma by influencing GLI1 expression. Unexpectedly, circ-GLI1 suppression had no apparent effect on the expression of GLI1 in both A375 and B16 cells (Fig. 4d). Moreover, we conducted β-lactamase activity assay to evaluate Hedgehog/GLI1 activity. First, we confirmed that GLI regulatory β-lactamase reporter gene was reduced by GLI1 silence rather than GLI2 and GLI3, indicating that GLI1 in A375 and B16 cells were the regulator of β-lactamase gene (Supplementary Fig. 1b). Circ-GLI1 inhibition overtly abrogated the functional activity of GLI1 (Fig. 4e). In addition, we found that silence of circ-GLI1 had no impact on the mRNA and protein levels of GLI2 and GLI3 (Supplementary Fig. 1c). These data indicated that circ-GLI1 positively regulated the activity of Hedgehog/GLI1 pathway. Also, Hedgehog and Wnt/β-catenin pathways are closely correlated and intertwined^26^. Therefore, we detected whether circ-GLI1 regulated Wnt/β-catenin pathway as well. TOP/FOP-Flash assay showed that activity of Wnt/β-catenin was repressed under the transfection of sh/circ-GLI1#1. (Fig. 4e). Besides, we found that the mRNA and protein levels of the downstream targets of the two pathways that were related to metastasis and angiogenesis, including SOX2, MYC, VEGF and MMP2^27–30^, were all abated upon circ-GLI1 silence, whereas GLI1 and β-catenin were changed at protein level but not mRNA level (Fig. 4f). Of interest, Cyr61 expression was sharply decreased in A375 cells by the respective treatment of the inhibitor of either Hedgehog signaling (GDC-0449) or Wnt/β-catenin pathway (XAV-939), and the dual-inhibition of the two pathways further enhanced the suppression on Cyr61 expression (Fig. 4g). Meanwhile, similar results were also observed in B16 cells (Fig. 4h, i). In addition, we validated that respective treatment of LiCl (Wnt/β-catenin activator) or SAG (Hedgehog activator) induced Cyr61 expression in two melanoma cells. Also, activation of these two pathways respectively restored Cyr61 level that was reduced by circ-GLI1 silence, and co-treatment of LiCl and SAG further enhanced the restoration (Supplementary Fig. 1d). By and large, circ-GLI1 positively regulates Cyr61 in melanoma via its dual-activation on Hedgehog and Wnt/β-catenin pathways.

### Circ-GLI1 elevates GLI1 and β-catenin protein levels by interacting with p70S6K2

**Fig. 5:**
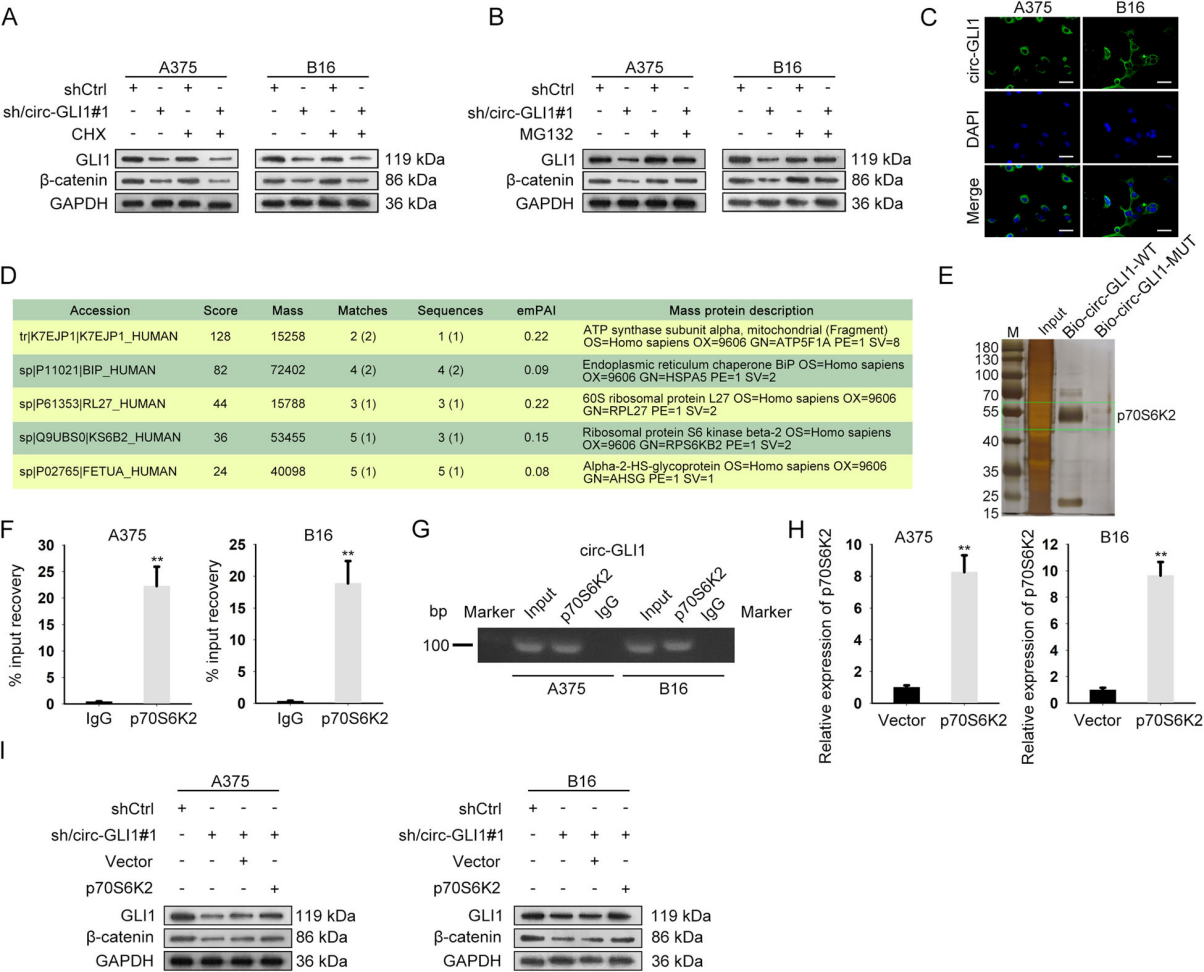
Circ-GLI1 modulates GLI1 and β-catenin via interacting with p70S6K2 in melanoma. **a**, **b** Western blot analysis of GLI1 and β-catenin protein in A375 and B16 cells with circ-GLI1 depletion lessened or together with CHX or MG132 treatment for 8 h. **c** FISH assay showed the main location of circ-GLI1 in cytoplasm. Scale bar = 100 μm. **d**, **e** RNA pull-down and mass spectrometry assays determined the proteins interacting with circ-GLI1. **f**, **g** RIP assay (Student’s *t* test) and nucleic acid electrophoresis indicated the interactivity of circ-GLI1 with p70S6K2. **h** qRT-PCR result of p70S6K2 level in A375 and B16 cells after transfected with overexpression vector. Student’s *t* test. **i** Western blot analysis uncovered that GLI1 and β-catenin protein levels were reduced by sh/circ-GLI1 but recovered partly by pcDNA3.1/p70S6K2. All data were obtained from at least three replicates and shown as mean ± SD. ^**^P < 0.01.

We proceeded to explore the mechanism by which circ-GLI1 regulated GLI1 and β-catenin. First of all, the cycloheximide (CHX, a protein synthesis inhibitor) and MG132 (proteasome inhibitor) were then used to treat A375 and B16 cells. Intriguingly, it appeared that the repressing effect of circ-GLI1 depletion on protein levels of both GLI1 and β-catenin was unchanged under CHX treatment but was evidently reversed in the context of MG132 application (Fig. 5a, b, Supplementary Fig. 2a, b), indicating that circ-GLI1 affected the expressions of GLI1 and β-catenin s in melanoma at post-translational level.

During past decades, circRNAs have been largely suggested to have different functions due to the varied subcellular localization, and they usually modulate gene expressions in cancer via interacting with certain RNAs or proteins. Therefore, we detected the distribution of circ-GLI1 in melanoma cells. Consequently, the FISH assay depicted the circ-GLI1 was predominantly located in the cytoplasm of both A375 and B16 cells (Fig. 5c). Moreover, ATP5F1A, HSPA5, RPL27, RPS6KB2, and AHSG were found to be several proteins (highest score) interacting with circ-GLI1 (Fig. 5d, e). Given that RPS6KB2 (also named p70S6K2) can affect GLI1 degradation by regulating GSK3β phosphorylation^31^, we payed attention on the interaction between p70S6K2 and circ-GLI1. As expected, the RIP assay validated a remarkable harvest of circ-GLI1 in anti-p70S6K2-immunoprecipitated compounds, while such enrichment was further proven by agarose gel electrophoresis (Fig. 5f, g). Furthermore, we testified that overexpression of p70S6K2 reversed the suppression of circ-GLI1 silence on the protein levels of GLI1 and β-catenin in melanoma cells (Fig. 5h, i, Supplementary Fig. 2c). In addition, we verified that overexpressing p70S6K2 had no impact on the mRNA level of GLI1, CTNNB1, GLI2, and GLI3 (Supplementary Fig. 2d). Collectively, circ-GLI1 facilitates the protein levels of GLI1 and β-catenin in melanoma in a p70S6K2-mediated way.

### Circ-GLI1 interacts with p70S6K2 to impair the interaction of GSK3β with GLI1 and β-catenin by regulating GSK3β phosphorylation

**Fig. 6:**
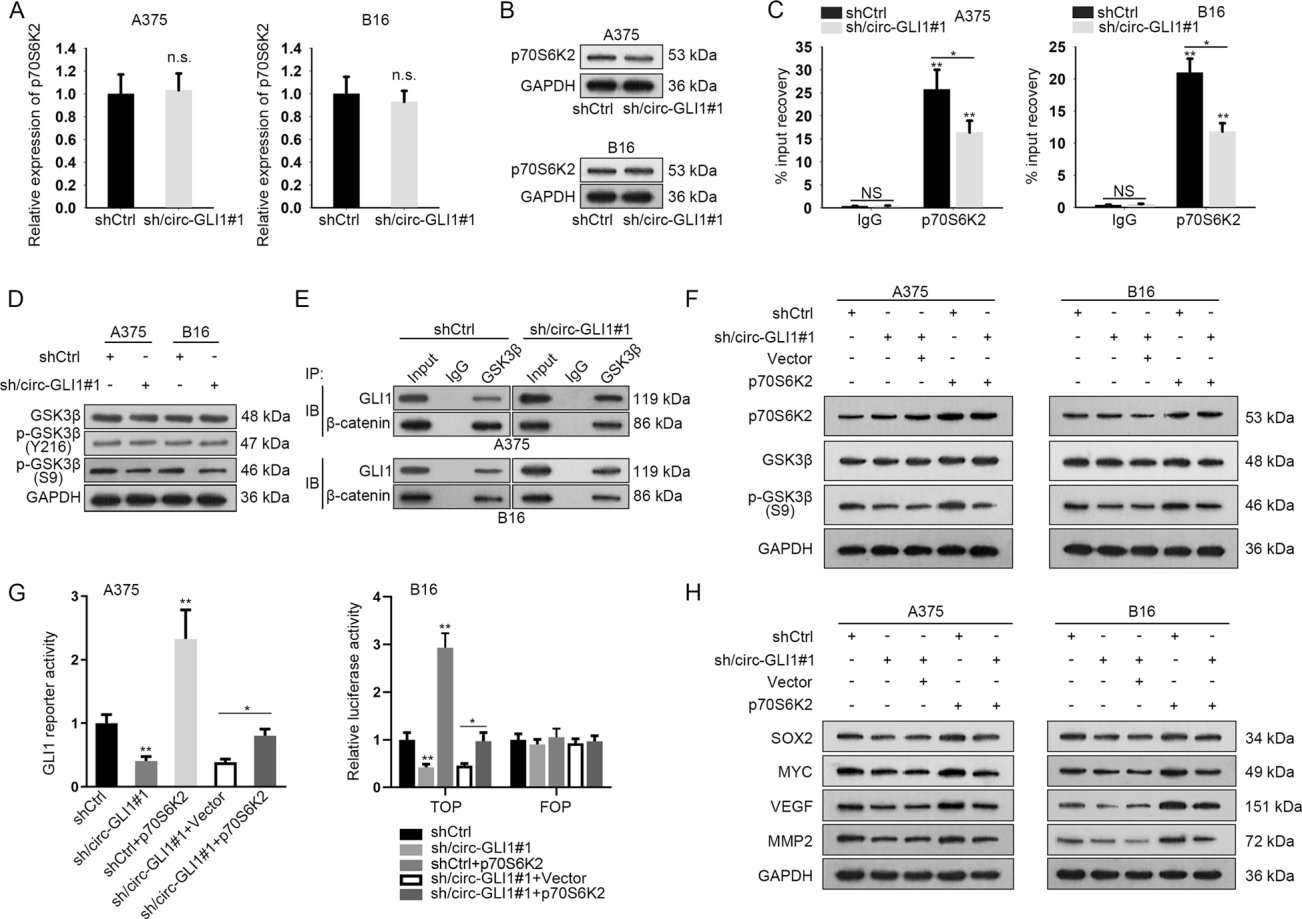
Circ-GLI1 regulates Hedgehog and Wnt/beta-catenin pathways by p70S6K2 in melanoma. **a**, **b** Both mRNA and protein levels of p70S6K2 were not affected by depleted circ-GLI1 at 48 h after transfection. Student’s *t* test. **c** RIP assay showed reduced interactivity between circ-GLI1 and p70S6K2 in melanoma cells under circ-GLI1 knockdown. Student’s *t* test. **d** The effect of circ-GLI1 inhibition on protein levels of total GSK3β, p-GSK3β (Y216), p-GSK3β (S9) was assessed by western blot. **e** Co-IP verified that circ-GLI1 affected the enrichment of GLI1 or β-catenin in GSK3β-precipitates. **f** The protein levels of p70S6K2, GSK3β (S9) and GSK3β were detected in cells transfected with sh/circ-GLI1 or p70S6K2 expression vector alone or co-transfected with sh/circ-GLI1 and p70S6K2 expression vector. **g** GLI1 reporter activity or TOP luciferase activity was assessed by luciferase reporter assay in cells transfected with shCtrl, sh/circ-GLI1#1, pcDNA3.1/p70S6K2 or sh/circ-GLI1#1 + pcDNA3.1/p70S6K2 for 48 h. One-way ANOVA. **h** Impact of p70S6K2 overexpression on the protein levels of SOX2, MYC, VEGF, and MMP2 in sh/circ-GLI1-mediated A375 and B16 cells was estimated by western blot. All data were obtained from at least three replicates and shown as mean ± SD. ^*^P < 0.05 and ^**^P < 0.01. n.s. indicated difference had no significance.

Previously, p70S6K2 has been demonstrated to enhance GLI1 protein via altering GSK3β phosphorylation at Ser9^31^. Therefore, we wondered whether circ-GLI1/p70S6K2 complex affected the expression of GLI1 and β-catenin in this manner. Both the mRNA and protein levels of p70S6K2 were not influenced by circ-GLI1 knockdown (Fig. 6a, b, Supplementary Fig. 3a). However, silencing circ-GLI1 in both A375 and B16 cells led to an obvious reduction on circ-GLI1-p70S6K2 interaction (Fig. 6c). In addition, circ-GLI1 inhibition resulted in an overt decline in the phosphorylation of GSK3β at Ser9 (the inactive form of GSK3β), with no impact on total GSK3β and GSK3β phosphorylation at Tyr216 (the active form of GSK3β) (Fig. 6d, Supplementary Fig. 3b). It is known that Ser9 phosphorylation deprived GSK3β from the binding with substrates at the catalytic site^32^. Concordantly, Co-IP results illustrated that circ-GLI1 depletion strengthened the interaction of GSK3β with either GLI1 or β-catenin in melanoma cells (Fig. 6e). Moreover, overexpression of p70S6K2 counteracted the suppressing impact of circ-GLI1 silence on phosphorylation of GSK3β at Ser9 (Fig. 6f, Supplementary Fig. 4a). Furtherly, the sh/circ-GLI1#1-caused reduction of Hedgehog/GLI1 and Wnt/β-catenin activities were reversed by p70S6K2 upregulation, which eventually led to the recovery of the levels of downstream targets (Fig. 6g, h, Supplementary Fig. 4b). In addition, overexpression of p70S6K2 increased the activities of GLI1 reporter and Wnt/β-catenin pathway. Meanwhile, the protein levels of the downstream targets of Wnt/β-catenin pathway were all enhanced a lot in response to p70S6K2 overexpression. By and large, these data highlighted that circ-GLI1 interacts with p70S6K2 to improve the stabilization of GLI1 and β-catenin proteins by phosphorylating GSK3β at Ser9.

### Circ-GLI1 enhances c-MYC-activated Cyr61 in melanoma through Hedgehog/GLI1 and Wnt/β-catenin pathways

**Fig. 7:**
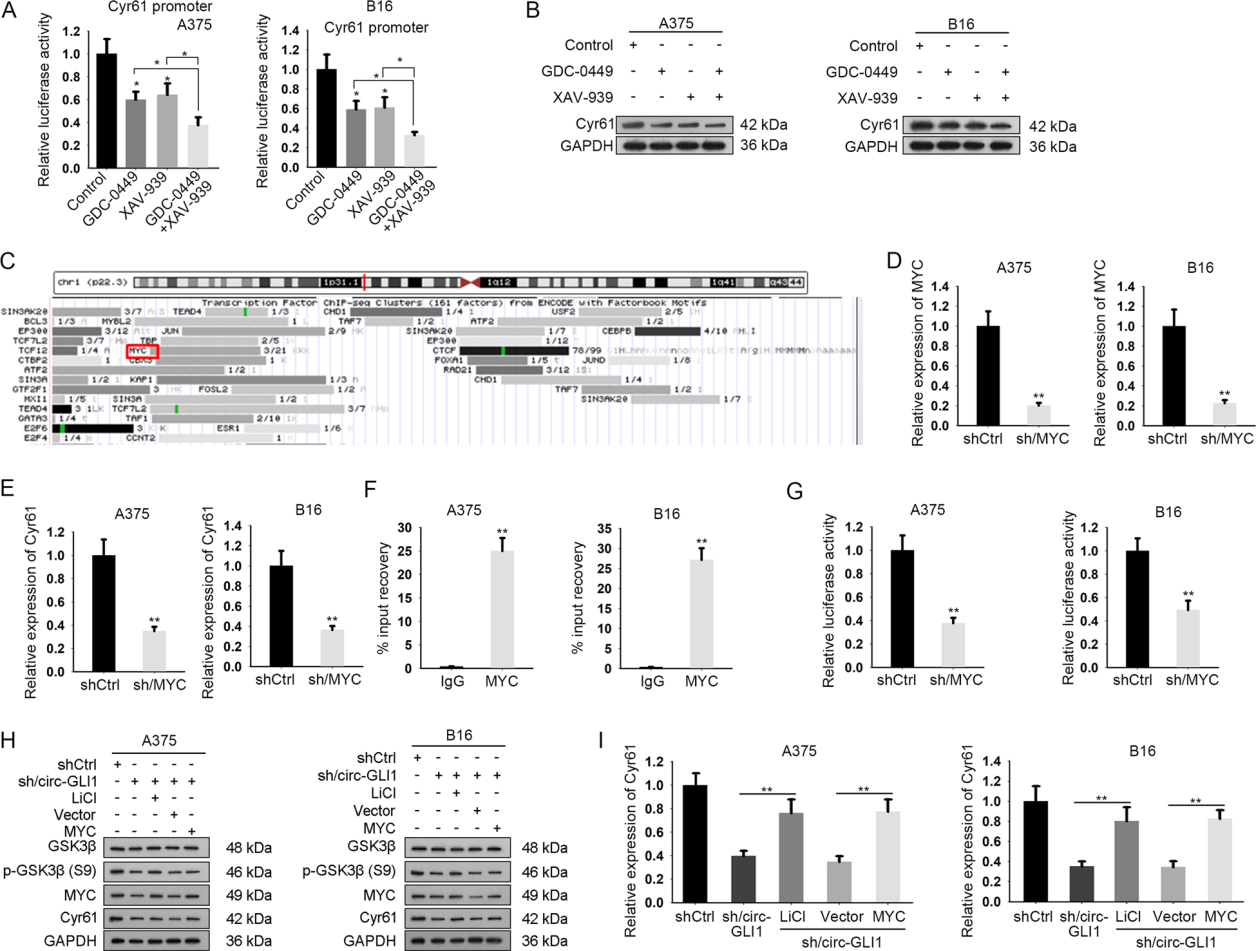
Circ-GLI1 activates Cyr61 by upregulating MYC in melanoma. **a**, **b** After treated for 24 h, GDC-0449, XAV-939 or both lowered Cyr61 promoter luciferase activity and Cyr61 protein level in A375 and B16 cells by conducting luciferase reporter assay and western blot. One-way ANOVA. **c** MYC was predicted as the potential transcriptional factor of Cyr61 by UCSC. **d**, **e** qRT-PCR suggested that sh/MYC transfection inhibited MYC and Cyr61 expressions in melanoma cells. Results were obtained at 48 h after transfection. Student’s *t* test. **f** ChIP assay testified the enrichment of Cyr61 promoter in anti-MYC-precipitates. Student’s t test. **g** MYC depletion lessened CYR61 promoter activity. **h** The protein levels of p-GSK3β (S9), MYC, Cyr61 in indicated melanoma cells were determined by western blot. Student’s *t* test. **i** qRT-PCR revealed the mRNA level of Cyr61 were strengthened by LiCl treatment, or upregulated MYC excepting p-GSK3β (S9). One-way ANOVA. All data were obtained from at least three replicates and shown as mean ± SD. ^*^P < 0.05 and ^**^P < 0.01.

Previously, we identified that circ-GLI1 promoted Cyr61 expression at transcriptional level through an indirect manner and that Cyr61 expression was affected by Hedgehog/GLI1 and Wnt/β-catenin pathways. Hence, we deduced that circ-GLI1 regulated Cyr61 transcription through Hedgehog/GLI1 and Wnt/β-catenin pathways. As expected, the activity of Cyr61 promoter and Cyr61 protein level were reduced when inhibiting either Hedgehog/GLI1 or Wnt/β-catenin pathway, and such effect was further decreased under the co-inhibition of the two pathways (Fig. 7a, b, Supplementary Fig. 4c). Intriguingly, UCSC data suggested that MYC potentially bound to Cyr61 promoter (Fig. 7c). MYC is a well-recognized transcription factor in the downstream the two pathways, and we have proved that circ-GLI1 positively regulated MYC expression by activating Hedgehog and Wnt/β-catenin. Hence, we deduced that circ-GLI1 activated Hedgehog and Wnt/β-catenin signaling to induce MYC, so as to trigger Cyr61 transactivation. Expectedly, Cyr61 expression was decreased along with MYC downregulation in melanoma cells (Fig. 7d, e). Meanwhile, we certified a high enrichment of Cyr61 promoter in anti-MYC group and that downregulation of MYC hindered the luciferase activity of Cyr61 promoter (Fig. 7f, g). Furthermore, it was proven that activation of Wnt/β-catenin pathway could increase the levels of p-GSK3β (Ser9), MYC and Cyr61, while MYC overexpression elevated the levels of MYC and Cyr61 in melanoma cells with circ-GLI1 silence (Fig. 7h, Supplementary Fig. 4d). Besides, the mRNA level of Cyr61 was also increased by inducing GSK3β phosphorylation (Ser9) using LiCl or MYC expression vector (Fig. 7i). In addition, Hedgehog pathway activator SAG had similar effect with LiCl (Supplementary Fig. 5a, b). However, treatment with the antagonist of Wnt/β-catenin pathway (DKK1) or Hedgehog pathway (Cyclopamine) had the opposite effects (Supplementary Fig. 5c–f). Thus, we concluded that circ-GLI1 contributes to Cyr61 transcription via MYC-mediated manner via Hedgehog/GLI1 and Wnt/β-catenin pathways.

### Circ-GLI1 facilitates metastasis and angiogenesis through Cyr61 in melanoma in vivo

**Fig. 8:**
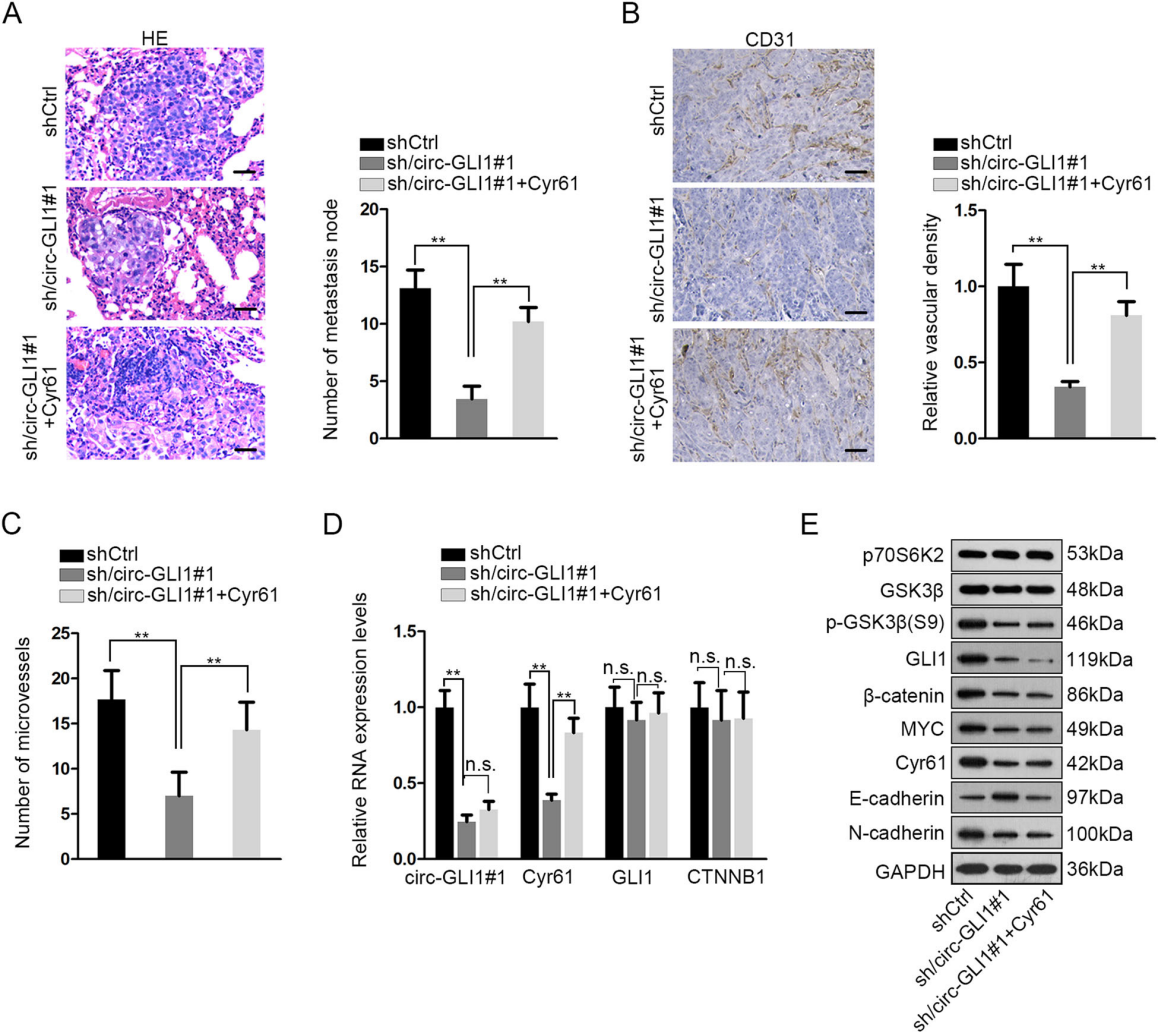
Circ-GLI1 facilitates metastasis and angiogenesis through Cyr61 in melanoma in vivo. **a** A375 cells were transfected with shCtrl, sh/circ-GLI1#1, or sh/circ-GLI1#1 + CYR61 respectively. The transfected cells were injected into nude mice from tail vain and grown for 32 days to investigate metastasis in vivo. Representative pictures and quantification of HE staining of metastatic nodules of each group. Scale bar = 100 μm; one-way ANOVA. **b** Transfected A375 cells were subcutaneously injected into nude mice and incubated for 28 days to detect angiogenesis. Representative images of IHC staining of CD31 in xenografts and quantification of relative vascular density of each group. Scale bar = 100 μm; one-way ANOVA. **c** Number of microvessels was evaluated in tumors of each group. One-way ANOVA. **d**. RT-qPCR data of the levels of circ-GLI1, Cyr61, GLI1, and CTNNB1 in tumors of each group. One-way ANOVA. **e** Western blots of p70S6K2, GSK3β, p-GSK3β, GLI1, β-catenin, MYC, Cyr61, E-cadherin, and N-cadherin in tumors of each group. All data were obtained from at least three replicates and shown as mean ± SD. ^**^P < 0.01.

Finally, we tested whether circ-GLI1 regulated Cyr61 to influence melanoma metastasis and angiogenesis in vivo. A375 cells stably transfected with shCtrl, sh/circ-GLI1#1, sh/circ-GLI1#1 + Cyr61 were injected into nude mice through tail vain. HE staining presented that silencing circ-GLI1 in A375 cells led to the reduction of metastatic nodules in vivo, but such reduction was reversed by the co-transfection by pcDNA3.1/Cyr61 (Fig. 8a). To monitor angiogenesis in vivo, transfected A375 cells were subcutaneously injected into the nude mice. IHC staining of CD31 decreased by circ-GLI1 silence in vivo, and such result was reversed by Cyr61 overexpression (Fig. 8b). The number of microvessels in tumors derived from A375 cells were reduced under circ-GLI1 silence and such effect was countervailed by the overexpression of Cyr61 (Fig. 8c). In addition, we validated that the levels of circ-GLI1 and Cyr61 decreased in tumors derived from A375 cells transfected with sh/circ-GLI1#1 in vivo, and only the decrease of Cyr61 was reversed by Cyr61 overexpression, confirming that circ-GLI1 was upstream regulator of Cyr61 (Fig. 8d). Meanwhile, the mRNA levels of GLI1 and CTNNB1 remained to be unchanged (Fig. 8d). Western blot results in Fig. 8e depicted that levels of p70S6K2 and GSK3β showed no significant variation among three groups. Levels of p-GSK3β (S9), GLI1, β-catenin, and MYC were downregulated by circ-GLI1 silence in vivo, but Cyr61 overexpression could not affect such downregulation. The level of Cyr61 was reduced by circ-GLI1 silence and restored by Cyr61 overexpression. In addition, circ-GLI1 silence led to the increased level of E-cadherin but the reduced level of N-cadherin in xenografts, and such effect was counteracted by Cyr61 upregulation. Taken, together, circ-GLI1 facilitated metastasis and angiogenesis through Cyr61 in vivo.

## Discussion

In the past few decades, scientists have discovered many molecules that participate in the initiation and progression of cancers. Cyr61 is a recently identified angiogenesis-related gene that has been found to function in many cancer types, including colorectal cancer^10^, multiple myeloma^33^, prostate cancer^34^. Of interest, the role of Cyr61 in melanoma seemed to be complex^14,16^. In the present study, we found Cyr61 contributed to melanoma cell migration, invasion and angiogenesis. This finding was consistent with our previous observation that Cyr61 involved in melanoma metastasis through regulating VEGF and MMP9^35^.

NcRNAs, a family of RNA transcripts lacking in protein products, are recognized as novel regulators in the development of a vast number of human diseases including cancer^20^. CircRNAs, members of the ncRNA family, have become a new research hot-spot^36^. Here, we screened out a novel circRNA named has_circ_0027247 (circ-GLI1) in melanoma. Circ-GLI1 was upregulated with highest fold change and had a potential to regulate Cyr61 in melanoma cells. Besides, the promoting effect of circ-GLI1 on melanoma metastasis was identified here for the first time. More importantly, we uncovered that circ-GLI1 modulated Cyr61 expression in melanoma cells through indirectly activating Cyr61 transcription.

CircRNAs usually exert functions by affecting the expression of adjacent genes, especially their host genes^37–39^. Therefore, we also evaluated whether circ-GLI1 affected GLI1 expression in melanoma. However, results showed that circ-GLI1 had no impact on GLI1 mRNA expression, but positively regulated the transcription activity of GLI1. GLI1 is the terminal effector molecule in Hedgehog signaling^25^, and we additionally validated that circ-GLI1 had no impact on GLI2 and GLI3. Therefore, we suggested that circ-GLI1 regulated Hedgehog/GLI1 pathway. Hedgehog/GLI1 signaling is widely known to show crosstalk with Wnt/β-catenin pathway^26^, so we further suggested that circ-GLI1 also regulated Wnt/β-catenin pathway. Interestingly, we demonstrated circ-GLI1 activated both Hedgehog/GLI1 and Wnt/β-catenin pathways by altering the expression of GLI1 and β-catenin at protein level but not mRNA level. In addition, the strong relationship between Hedgehog/GLI1 and Wnt/β-catenin in malignancies has already been unveiled^40,41^. Furthermore, we found that circ-GLI1 modulated GLI1 and β-catenin at post-translational level by influencing their degradation.

CircRNAs can regulate gene expressions by interacting with proteins or RNAs^42^. In the present study, five RBPs including ATP5F1A, HSPA5, RPL27, RPS6KB2, and AHSG were screened out due to their highest enrichment score, among which, RPS6KB2 (namely p70S6K2) can repress GSK3β activity by phosphorylating Ser9 residue^31^. Ser9 phosphorylation at N-terminal domain of GSK3β serves as a pseudo-substrate to prevent substrates from binding to the catalytic site^32^, while un-phosphorylated GSK3β can interact with β-catenin through adenomatous polyposis coli gene (APC) β-catenin complex to trigger its phosphorylation, and can catalyze PKA-primed phosphorylation to induce GLI1 degradation^43,44^. Current study validated that circ-GLI1 interacted with p70S6K2 to inactivate GSK3β by phosphorylating GSK3β at Ser9, which blocked the binding of GSK3β to GLI1 and β-catenin and therefore inhibited degradation and enhanced expression of the two proteins.

MYC is a well-recognized downstream target of β-catenin^45^ and can also be modulated by GLI1 signaling^46,47^. In the meantime, MYC is a transcription factor that modulates gene expression during tumorigenesis^48^. In this study, UCSC indicated MYC as a potential transcription factor of Cyr61, and the positive regulation of MYC on Cyr61 transcription was further certified here. In addition, we illustrated that circ-GLI1 activated Hedgehog/GLI1 and Wnt/β-catenin pathways to induce MYC-regulated transactivation of Cyr61 in melanoma. We also testified that Cyr61 was the responsible mediator for circ-GLI1-contributed melanoma metastasis and angiogenesis both in vitro and in vivo. Intriguingly, reports also revealed that MYC could conversely modulate GLI1 transcription^49,50^, suggesting a feedback loop of MYC-GLI1 in melanoma.

## Conclusion

In conclusion, our research unveiled that circ-GLI1 interacted with p70S6K2 to boost GLI1 and β-catenin proteins, so as to activate Hedgehog/GLI1 and Wnt/β-catenin pathways and therefore enhance MYC-activated Cyr61 expression, leading to accelerated metastasis in melanoma. These findings provided strong evidence that circ-GLI1 could be a novel promising therapeutic target to relieve melanoma metastasis.

## Supplementary information

Supplementary figure legends

Supplementary Table

Figure S1

Figure S2

Figure S3

Figure S4

Figure S5
