# Supplementary material for: Circ-GLI1 promotes metastasis in melanoma through interacting with p70S6K2 to activate Hedgehog/GLI1 and Wnt/β-catenin pathways and upregulate Cyr61
Source: Cell Death Dis. 2020 Jul 30;11(7):596. doi: 10.1038/s41419-020-02799-x (PMC7393080; doi:10.1038/s41419-020-02799-x)
Supplement: Supplementary file 1 — Supplementary figure legends [file 41419_2020_2799_MOESM1_ESM.docx]

**Supplementary figure legends**

**Supplementary figure 1** A. qRT-PCR data of the knockdown efficiency of circ-GLI1 by sh/circ-GLI1#1/2 in A375 and B16 cells. One-way ANOVA. B. GLI regulatory β-lactamase activity under the silence of GLI1, GLI2, GLI3, and negative control PLK1. One-way ANOVA. C. qRT-PCR analysis of GLI2 and GLI3 under circ-GLI1 silence. Student’s t test. D. Melanoma cells were transfected with shCtrl or sh/circ-GLI1#1, and treated with LiCl or SAG, the activator of Wnt/β-catenin or Hedgehog pathways. qRT-PCR data of Cyr61 level in melanoma cells of indicated groups. One-way ANOVA. All data were obtained from at least three replicates and shown as mean ± SD. The asterisk (^*^) means P < 0.05 and (^**^) indicates P < 0.01.

**Supplementary figure 2** A-C. Quantification of western blot in Figure 5A, 5B and 5I. One-way ANOVA. D. Melanoma cells were transfected with pcDNA3.1 vectors or pcDNA3.1/p70S6K2. qRT-PCR analysis of GLI1, GLI2, GLI3 and CTNNB1 in melanoma cells of each group. Student’s t test. All data were obtained from at least three replicates and shown as mean ± SD. The asterisk (^**^) indicates P < 0.01. n.s. indicated difference had no significance.

**Supplementary figure 3** A-B. Quantification of western blot in Figure 6B and 6D. Student’s t test. All data were obtained from at least three replicates and shown as mean ± SD. The asterisk (^**^) indicates P < 0.01. n.s. indicated difference had no significance.

**Supplementary figure 4** A-D. Quantification of western blot in Figure 6F, 6H, 7B, and 7H. All data were obtained from at least three replicates and shown as mean ± SD. One-way ANOVA. The asterisk (^*^) means P < 0.05 and (^**^) indicates P < 0.01. n.s. indicated difference had no significance.

**Supplementary figure 5** A. The protein levels of p-GSK3β (S9), MYC, Cyr61 in circ-GLI1-downregulated melanoma cells were detected by western blot after treatment with Hedgehog pathway activator SAG or MYC expression vector. B. qRT-PCR analysis of Cyr61 mRNA in circ-GLI1-downregulated melanoma cells after treated with Hedgehog pathway activator SAG or MYC expression vector. One-way ANOVA. C. The protein levels of p-GSK3β (S9), MYC, Cyr61 in circ-GLI1-downregulated melanoma cells were detected by western blot after treatment with WNT pathway antagonist DKK1 or MYC expression vector. D. qRT-PCR analysis of Cyr61 mRNA in circ-GLI1-downregulated melanoma cells after treated with WNT pathway antagonist DKK1 or MYC expression vector. One-way ANOVA. E. The protein levels of p-GSK3β (S9), MYC, Cyr61 in circ-GLI1-downregulated melanoma cells were detected by western blot after treatment with Cyclopamine or MYC expression vector. F. qRT-PCR analysis of Cyr61 mRNA in circ-GLI1-downregulated melanoma cells after treated with Cyclopamine or pcDNA3.1/MYC. One-way ANOVA. All data were obtained from at least three replicates and shown as mean ± SD. The asterisk (^*^) means P < 0.05 and (^**^) indicates P < 0.01.
