# Supplementary figures and images for: Circ-GLI1 promotes metastasis in melanoma through interacting with p70S6K2 to activate Hedgehog/GLI1 and Wnt/β-catenin pathways and upregulate Cyr61
Source: Cell Death Dis. 2020 Jul 30;11(7):596. doi: 10.1038/s41419-020-02799-x (PMC7393080; doi:10.1038/s41419-020-02799-x)

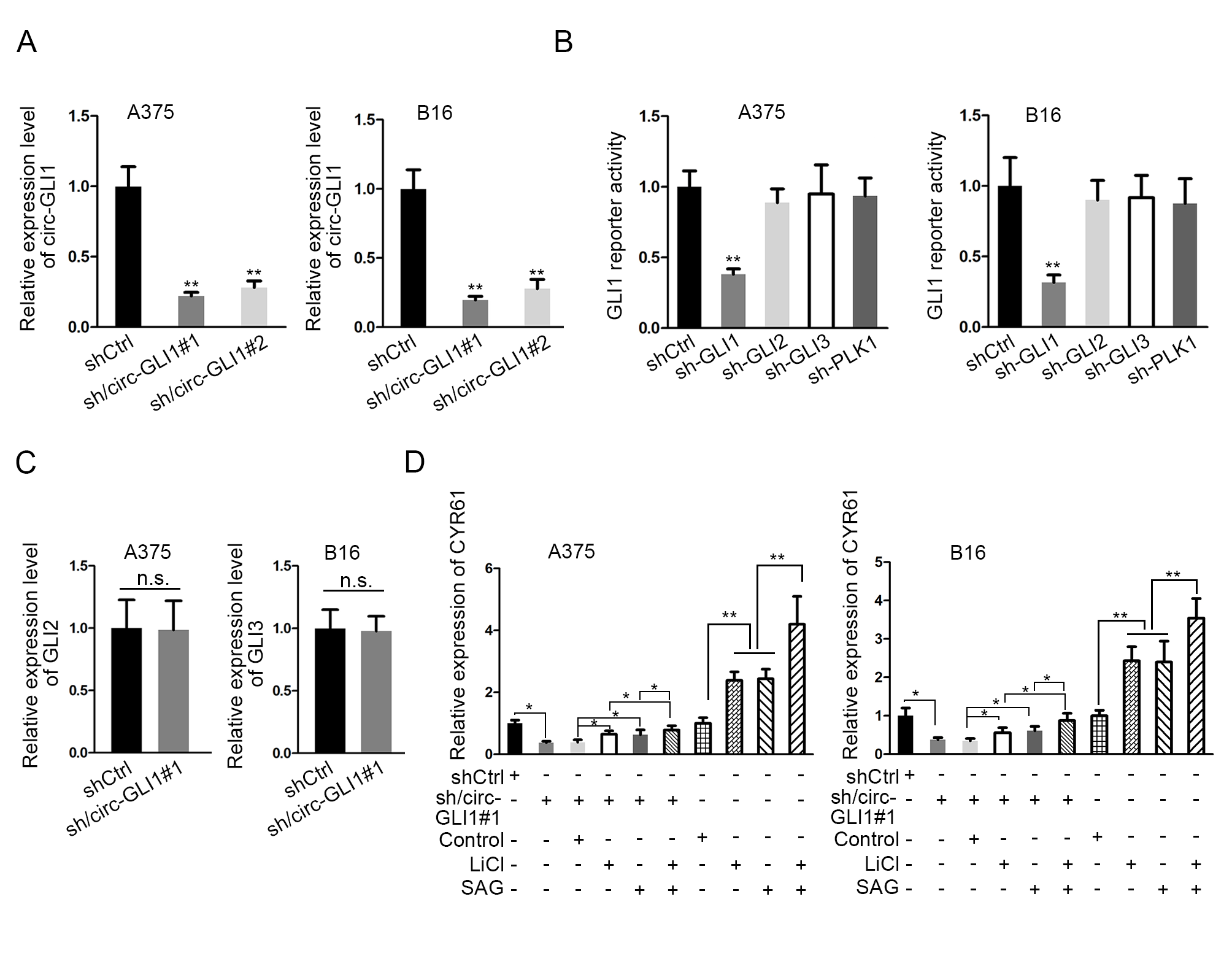

Supplement: Supplementary file 3 — Figure S1 [file 41419_2020_2799_MOESM3_ESM.tif]

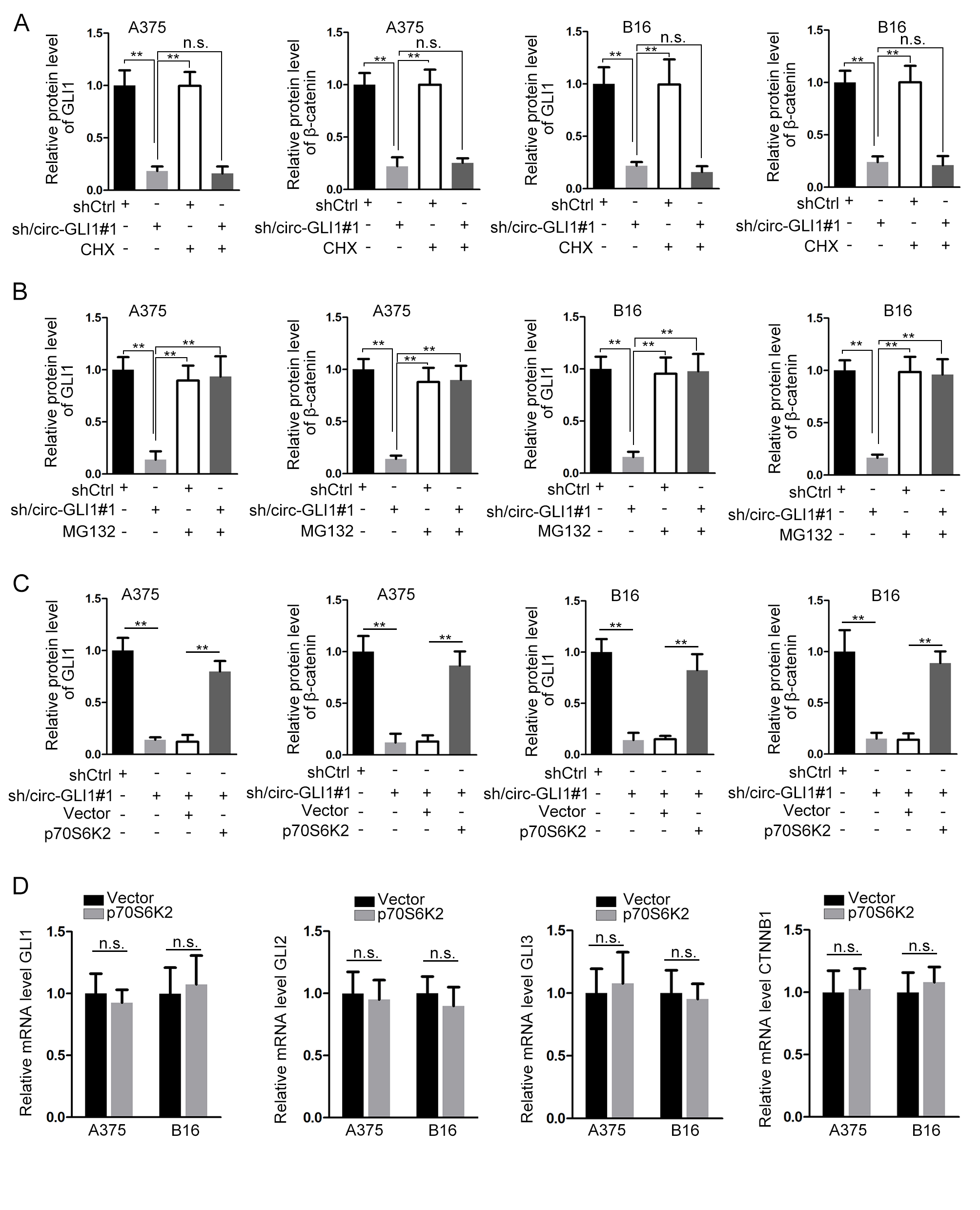

Supplement: Supplementary file 4 — Figure S2 [file 41419_2020_2799_MOESM4_ESM.tif]

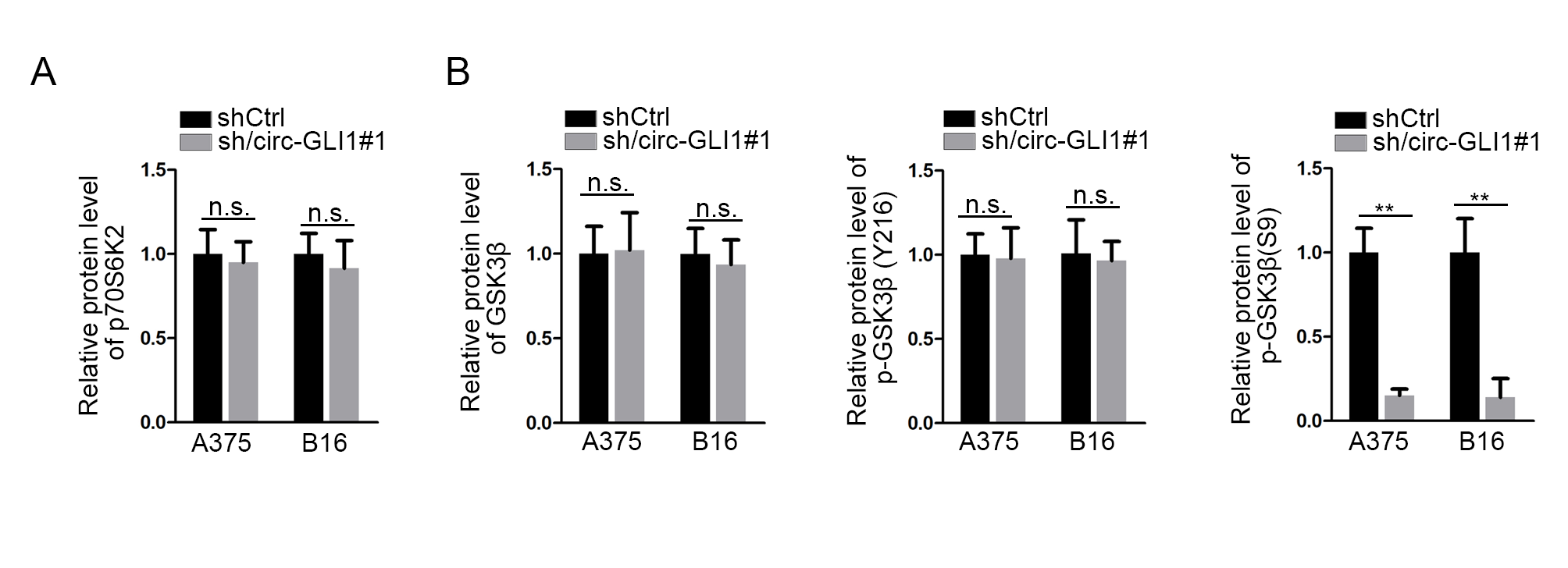

Supplement: Supplementary file 5 — Figure S3 [file 41419_2020_2799_MOESM5_ESM.tif]

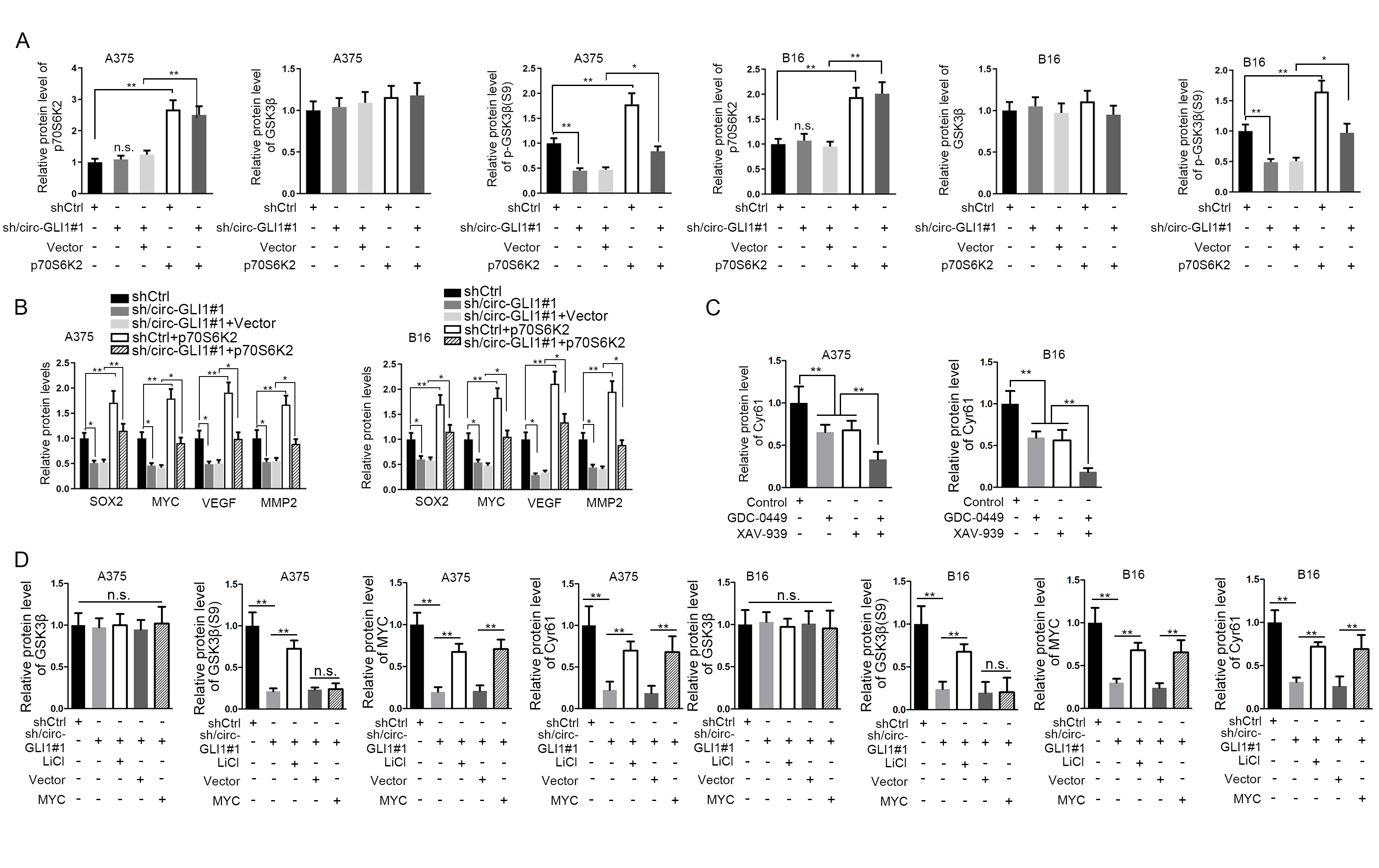

Supplement: Supplementary file 6 — Figure S4 [file 41419_2020_2799_MOESM6_ESM.tif]

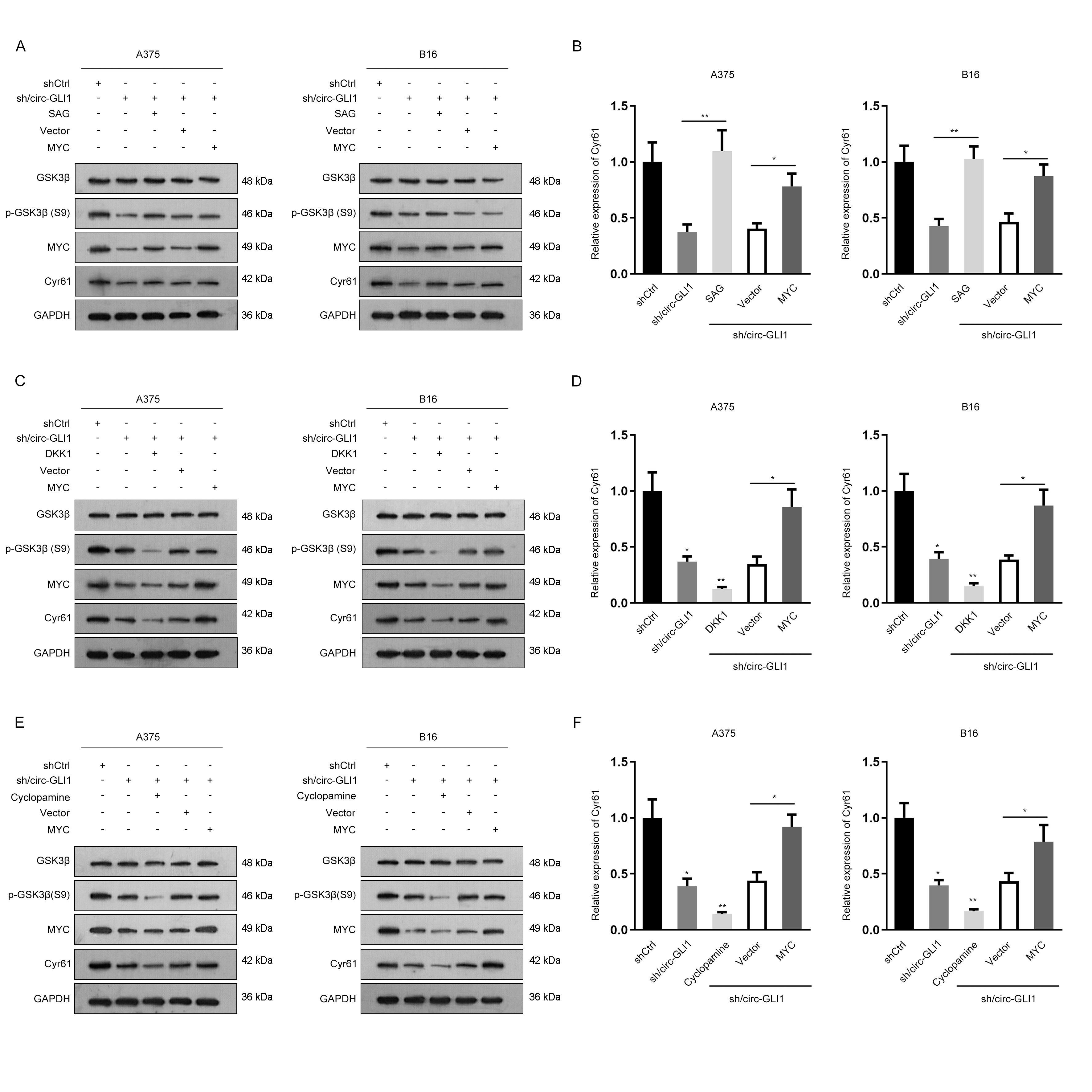

Supplement: Supplementary file 7 — Figure S5 [file 41419_2020_2799_MOESM7_ESM.tif]
